# SARS-CoV-2 infection in China—Before the pandemic

**DOI:** 10.1371/journal.pntd.0008472

**Published:** 2020-08-06

**Authors:** Huiying Liang, Lingling Zheng, Huimin Xia, Jinling Tang

**Affiliations:** 1 Department of Clinical Data Center, The Guangzhou Women and Children’s Medical Center, Guangzhou Medical University, Guangzhou, China; 2 The Guangdong Provincial Children's Medical Research Center, Guangzhou, China; 3 The Guangdong Provincial Key Laboratory of Research in Structural Birth Defect Disease, Guangzhou, China; 4 Department of the School of Public Health and Primary Care, Chinese University of Hong Kong, Hong Kong, China; London School of Hygiene & Tropical Medicine, UNITED KINGDOM

## Abstract

In order to rapidly inform polices in the international response to the ongoing pandemic of coronavirus disease 19 (COVID-19), we summarize in this review current evidence on epidemiological and clinical features of the infection, transmission routes, problems of nucleic-acid testing, the epidemiological trend in China and impact of interventional measures, and some lessons learned. We concluded that the epidemic is containable with traditional nonpharmacological interventions, mainly through social distancing and finding and isolating suspected patients and close contacts. Nonpharmacological interventions are the only effective measures currently accessible and have suppressed some 90% of the infections in China. Close contacts are the major mechanism of transmission, which makes it possible to control this epidemic through nonpharmacological methods. Nucleic-acid testing alone may miss some 50% of infected patients, and other methods such as chest computerized tomography (CT) or serology should be considered to supplement molecular testing. The development of vaccines and drugs is important, but hesitation to make use of nonpharmacological interventions may mean missing golden opportunities for effective actions.

## Author summary

In particular, we summarize the origin of the pathogen, epidemiological and clinical features of the infection, transmission routes and transmissibility, problems of nucleic-acid testing and diagnostic criteria, the epidemiological trend in China and its relation to interventional measures, and special issues including infection in doctors and children and the role of vaccines versus traditional nonpharmacological interventions in control of the epidemic. Importantly, evidence clearly suggests the following:

1. The epidemic is containable with traditional nonpharmacological interventions, largely by social distancing and testing and isolation of suspected patients and close contacts.
2. Nonpharmacological interventions are the most immediately accessible effective measures and have helped suppress some 90% of infections in China.
3. Close contacts through droplets and fomites are the major mechanism of transmission, and asymptomatic infections and aerosols are unlikely important in spreading the virus. This is a major feature of the virus and explains why it is containable through nonpharmacological methods.
4. Nucleic-acid testing alone may miss many truly infected patients, and chest CT should always be used if available to supplement molecular testing.
5. Development of vaccines and drugs are important, but any hesitation to use nonpharmacological interventions may mean missing golden opportunities for useful actions against the epidemic.

## Introduction

In December 2019, a series of patients with pneumonia of unknown etiology were noted in Wuhan, Hubei Province, China [1,2]. It was soon confirmed to be a highly contagious infectious disease caused by a new virus now known as Severe Acute Respiratory Syndrome Coronavirus 2 (SARS-CoV-2), which is similar to the coronavirus responsible for Severe Acute Respiratory Syndrome (SARS) [3,4]. Before March 2020, the epidemic was largely confined to China [5], and massive measures were taken to combat it [6]. It is now steadily abating in China but growing at a considerable pace outside, forming a worldwide pandemic inflicting over 180 countries [5]. The total number of cases reported outside China has surpassed China on 16 March, increasing at a rate of 20% daily in the past 30 days in developed countries [5]. We summarized the epidemiological and clinical features of the disease and control measures and their impact in China with the objective of informing international planning on next steps in the response to the ongoing epidemic.

## Pathogen

The pathogen of the pneumonia was confirmed on January 7, 2020 to be a new human-infecting coronavirus [2–4,7], which was first named 2019 novel coronavirus (2019-nCoV) by the World Health Organization [8] and SARS-CoV-2 as preferred by the International Committee on Taxonomy of Viruses [9]. Genetically, 2019-nCoV belongs to the coronavirus family, which includes SARS and Middle East Respiratory Syndrome (MERS) [2] but is more contagious and less lethal than the latter two [10,11]. The virus was found to be 79.6% genetically identical to SARS-CoV and 96.2% to a bat coronavirus detected in *Rhinolophus affinis*, a likely reservoir of SARS-CoV-2 [3,7].

Investigations into the intermediate hosts and original epidemic center are inconclusive. Pangolin, a wild animal, was likely an intermediate host responsible for passing the virus from bats to humans, from which a corona virus 91% identical to SARS-CoV-2 at the whole genome level [12] and 93% identical at the spike glycoprotein protein sequences was isolated [13]. Animal–human transmission likely occurred in the Huanan Seafood Wholesale Market in Wuhan, for which 27 (65.9%) of the first 41 coronavirus disease 19 (COVID-19) cases had a history of direct exposure [14]. In addition, the observation that 33 out of 585 environmental samples collected from the market were positive for the nucleic acid of 2019-nCoV further supports this hypothesis.[15] However, the very first patient recorded December 1, 2019 and many early patients did not have any relation to the market, raising questions as to whether the virus originated from there [14,16].

## Epidemiology

COVID-19 was highly contagious. The basic reproduction number (*R*_*0*_), the average number of cases a patient can infect during the entire infectious period and in the absence of intervention, is a commonly used measure for the contagiousness of infectious diseases. By using data before January 20, 2020 when no intervention measures were widely taken in China, *R*_*0*_ for 2019-nCoV was estimated around 3.3 (ranging from 1.4–6.5) [17], as compared with 2.7 (2.0–4.0) for SARS [18,19] and below 1 for MERS [20].

### Incubation period

**Fig 1:**
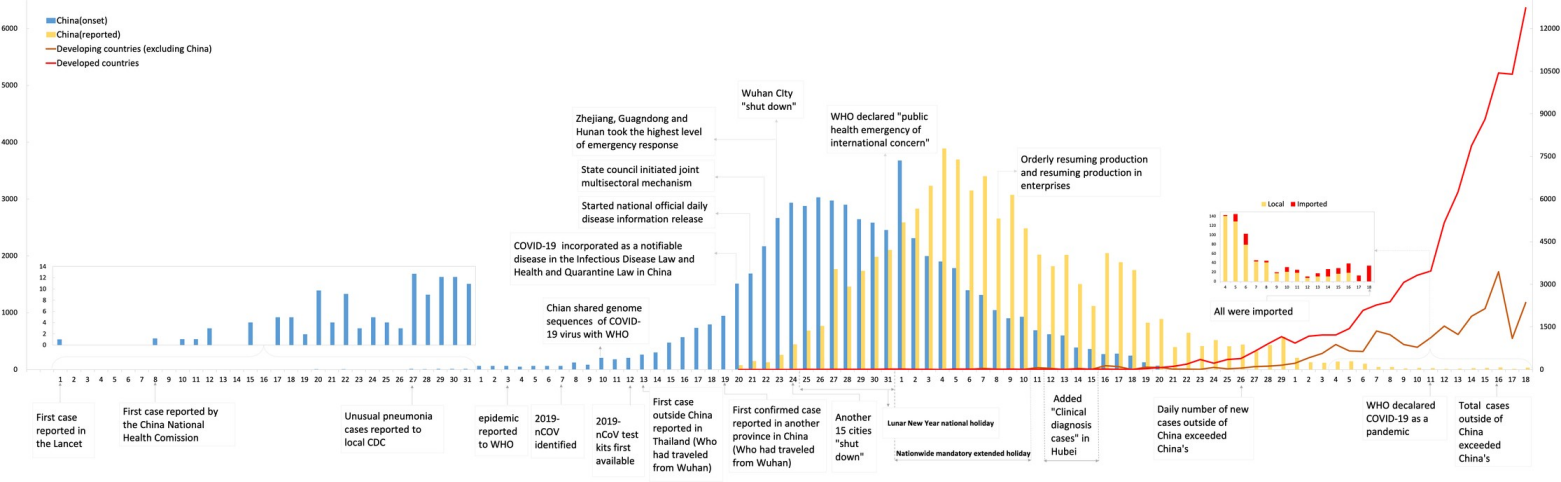
Approximate number of days from infection to onset, to first medical visit, to hospitalization, to laboratory diagnosis, and to discharge with COVID-19 patients. (Table 1 is referred for more details.) CDC, Centers for Disease Control and Prevention; COVID-19, coronavirus disease 19; 2019-nCoV, 2019 novel coronavirus.

The incubation period is approximately 6.0 days (Fig 1), varying from 4.0 to 7.4 days as reported in different studies (Table 1 [21–33]). Two weeks are usually used as the longest incubation time for substantiating diagnoses, tracing sources of close contacts, and quarantining suspected patients, although 1.0%–5.8% patients may have an incubation period over 14 days [34,35], which in some may be over 20 days [34,36,37].

**Table 1:** Incubation period, duration of hospitalization, and time from onset to medical visit, hospitalization, and laboratory diagnosis of COVID-19.

Authors	Location/Population	Period of Study	Case Numbers	Estimates (days)	Variation
**Incubation Period**					
Li and colleagues [21]	Wuhan	Before Jan 21	10	5.2^a^	95% CI 4.1–7.0
Xu and colleagues [22]	Zhejiang	Jan 10–26	56	4.0^b^	IQR 3.0–5.0
Backer and colleagues [23]	Travelers from Wuhan	Jan 20–28	88	6.4^a^	95% CI 5.6–7.7
Guan and colleagues [24]	China	Dec 11–Jan 29	291	4.0^b^	IQR 2.0–7.0
Linton and colleagues [25]	China, publicly available	Before Jan 31	158	5.6^c^	95% CI 5.0–6.3
Tian and colleagues [26]	Beijing	Before Feb 10	262	6.7	SD 5.2
Bi e and colleagues [27]	Shenzhen	Jan 14–Feb 12	183	4.8^b^^,^^d^	95% CI 4.2–5.4
Wang and colleagues [28]	Henan	Jan 21–Feb 14	483	7.4^d^	95% CI 2.0–20.0
WHO [6]	China	Before Feb 20	NA	5.0–6.0	Range 1–14
**Onset to first medical visit**					
Li and colleagues [21]	Wuhan	Before Jan 1	45	5.8^a^	95% CI 4.3–7.5
Li and colleagues [21]	Wuhan	Jan 1–11	207	4.6^a^	95% CI 4.1–5.1
Sun and colleagues [29]	China, publicly available	Before Jan 18	NA	5.0^b^	NA
Sun and colleagues [29]	China, publicly available	After Jan 18	NA	2.0^b^	NA
Sun and colleagues [29]	China, publicly available	Jan 22–30	200	3.0^b^	IQR 0.0–15.0
W u and colleagues [30]	Tianjin	Before Feb 18	40	4.5^b^	Range 1.0–13.0
**Onset to hospitalization**					
Huang and colleagues [14]	Wuhan	Dec 16–Jan 1	41	7.0^b^	IQR 4.0–8.0
Li and colleagues [21]	Wuhan	Before Jan 1	44	12.5^a^	95% CI 10.3–14.8
Li and colleagues [21]	Wuhan	Jan 1–11	189	9.1^a^	95% CI 8.6–9.7
Xu and colleagues [22]	Zhejiang	Jan 10–26	62	2.0^b^	IQR 1.0–4.3
Wang and colleagues [31]	Wuhan	Jan 1–28	138	7.0^b^	IQR 4.0–8.0
Linton and colleagues [25]	Outside of Wuhan, living	Before Jan 31	155	3.3^b^^,^^c^	95% CI 2.7–4.0
Linton and colleagues [25]	Outside of Wuhan, deceased	Before Jan 31	34	6.5^b^^,^^c^	95% CI 5.2–8.0
Tian and colleagues [26]	Beijing	Before Feb 10	262	4.5	SD 3.7
**Onset to laboratory diagnosis**					
WHO [6]	China	early Jan	NA	12.0^b^	Range 8.0–18.0
WHO [6]	China	early Feb	NA	3.0^b^	Range 1.0–7.0
WHO [6]	Wuhan	early Jan	NA	15.0^b^	Range 10.0–21.0
WHO [6]	Wuhan	early Feb	NA	5.0^b^	Range 3.0–9.0
Bi and colleagues [27]	Shenzhen	Jan 14–Feb 12	183	5.5	95% CI 5.0–5.9
**Hospitalization to laboratory diagnosis**					
Tian and colleagues [26]	Beijing	Before Feb 10	262	2.1	SD 1.9
**Onset to dyspnea**					
Huang and colleagues [14]	Wuhan	Dec 16–Jan 2	41	8.0^b^	95% CI 5.0–13.0
Wang and colleagues [31]	Wuhan	Jan 1–28	138	5.0^b^	IQR 1.0–10.0
**Onset to ICU admission**					
Wang and colleagues [31]	Wuhan	Jan 1–28	36	10.0	IQR 6.0–12.0
Zhou and colleagues [32]	Wuhan, ≥18 years old	Before Jan 31	191	12.0^b^	IQR 8.0–15.0
**Onset to clinical recovery**					
WHO [6]	China, mild cases	Before Feb 20	NA	NA	2.0 weeks
WHO [6]	China, severe cases	Before Feb 20	NA	NA	3.0–6.0 weeks
**Onset to death**					
Wang and colleagues [30]	China	Dec 1–Jan 26	17	14.0^b^	Range 6.0–41.0
Wang and colleagues [30]	China, 70 years old or above	Dec–Jan 26	NA	11.5	Range 6.0–19.0
Wang and colleagues [30]	China, below 70 years old	Dec 1–Jan 26	NA	20.0	Range 10.0–41.0
Linton and colleagues [25]	China, publicly available	Before Jan 31	158	15.0^d^	95% CI 12.8–17.5
WHO [6]	China	Before Feb 20	2,114	NA	Range 2.0–8.0 weeks
Zhou and colleagues [32]	Wuhan, ≥18 years old	Before Jan 31	54	18.5^b^	IQR 15.0–22.0
**Hospitalization to death**					
Linton and colleagues [25]	China, publicly available	Before Jan 31	16	8.8^a^	95% CI 7.2–10.8
Zhou and colleagues [32]	Wuhan, ≥18 years old	Before Jan 31	54	7.5^b^	IQR 5.0–11.0
**Hospitalization to discharge**					
Guan and colleagues [24]	China	Dec 11–Jan 29	963	12.0	IQR 10.0–14.0
Zhou and colleagues [32]	Wuhan, ≥18 years old	Before Jan 31	137	12.0^b^	IQR 9.0–15.0
Wu and colleagues [33]	Wuhan	Dec 25–Feb 13	201	13.0^b^	IQR 10.0–16.0

**Abbreviations:** COVID-19, coronavirus disease 19; ICU, intensive care unit; IQR, interquartile range; NA, not reported in the paper; 95% CI, 95% confidence interval.

^a^Weibull distribution was used to estimate means and 95% CI.

^b^The estimates are medians, the rest are means.

^c^Gamma distribution was used to estimate median and 95% credible interval.

^d^Log-normal distribution was used to estimate median and 95% credible interval.

In addition, the time from onset of illness to hospitalization (equivalent to the time quarantine starts) is the duration in which patients are infectious. It is crucial to reduce this time window for epidemic control. In China, it was on average 14 days before 14 January and reduced to 1 day after 22 January, indicating the considerable impact of measures implemented [4,21]. The average time from hospitalization to discharge is above 12 days [24,32,33], which is important for planning hospital resources, in particular during the peak period of the epidemic. For example, some 2,000 new patients a day needed to be hospitalized in Wuhan in the peak days.

Furthermore, there was an average of 4.5 days between patients’ first medical visit and time of hospitalization. This partly contributed to the delay from onset of illness to hospitalization. The 2-day delay in diagnosis after hospitalization was partly a result of insufficient supply of testing kits but would not cause spreading of virus because patients had already been under quarantine [26]. As a result, the date of public reporting was on average 9 days later than the date of illness onset in China (Fig 1).

### Epidemic trend

**Fig 2:**
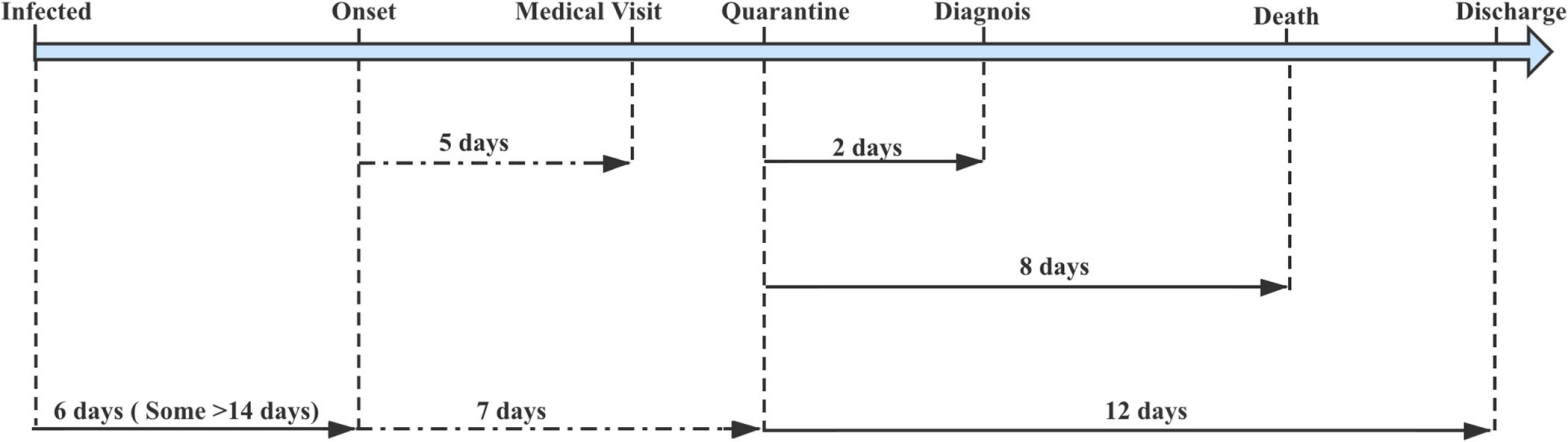
Epidemic curve of the confirmed cases of COVID-19 and time points of major related events. Daily numbers of confirmed cases are plotted in bars by the date of onset (blue) and by that of diagnosis/reporting (yellow). In the inset are cases in December 2019 (left) and after March 5, 2020 (right). Cases reported outside of China were separately plotted between western countries (red lines) and other countries (brown lines). The daily number of cases by the date of onset were adapted from the data in Fig 3 in the Report of the WHO–China Joint Mission on COVID-19 [6], the rest of the data are from China CDC [5], and the events are from various publications included in this paper. CDC, Centers for Disease Control and Prevention; COVID-19, coronavirus disease 19.

Fig 2 shows the chronological development of the epidemic and major related events between December 1, 2019 and March 18, 2020. The first case in China occurred December 1, 2019 [14], the first case outside China was diagnosed January 13, 2020 in Thailand, and the first case in another province of China was reported January 19, 2020. The epidemic was steadily escalating until January 20, 2020, before which no systematic, large-scale interventions were embarked upon.

January 20, 2020 marked the beginning of the massive combat against the epidemic in China. First of all, COVID-19 was incorporated into class-B notifiable diseases and treated as a class-A disease in action, which was followed by massive nationwide control efforts. Notably, Wuhan, the central epidemic area and a city of 11 million in population, was “locked down” and put under wholesale quarantine on January 23, and all the intercity and soon intracity transports were shut down [6]. More detailed measures are listed in Table 2, including notably identifying and isolating patients and their close contacts, social distancing, mask wearing, closures of public gatherings, quarantine of infected residential areas, and extending the Chinese New Year holidays. Since then, the entire country has come into a public health emergency response unprecedented in its recent history.

**Table 2:** Nationwide action against the epidemic in China^a^.

**The First Stage**
• Wet markets were closed, and efforts were made to identify the zoonotic source. • WHO was notified of information on the epidemic (Jan 3). • Whole genome sequences of the COVID-19 virus were shared with WHO (Jan 10). • Protocols for COVID-19 diagnosis and treatment, surveillance, epidemiological investigation, management of close contacts, and laboratory testing were formulated. • Relevant surveillance activities and epidemiological investigations were conducted. • Diagnostic testing kits were developed. • Wildlife and live poultry markets were placed under strict supervision and control measures.
**The Second Stage**
• Wildlife markets were closed. • Wildlife captive-breeding facilities were cordoned off. • COVID-19 was included in the statutory report of class-B infectious diseases and border health quarantine infectious diseases (Jan 20). • Temperature checks. • Healthcare declarations. • Quarantine at transportation deports. • Wuhan implemented strict traffic restrictions (Jan 23). • The protocols for diagnosis, treatment, and epidemic prevention and control were improved. • Case isolation and treatment were strengthened. • Extension of the Spring Festival holiday. • Traffic controls. • Control of transportation capacity to reduce the movement of people. • Cancellation of mass gathering activities. • Information about the epidemic and prevention and control measures was regularly released. • Public risk communications and health education were strengthened. • Allocation of medical supplies was coordinated. • New hospitals were built. • Reserve beds were used. • Relevant premises were repurposed to ensure that all cases could be treated. • Maintaining of a stable supply of commodities and prices to ensure the smooth operation of society.
**The Third Stage**
• Fully implement relevant measures for the testing, admission, and treatment of all patients for Wuhan and other priority areas of Hubei Province. • Relevant measures and new technologies were strengthened in the areas of epidemiological investigation, case management, and epidemic prevention in high-risk public places. • Relevant health insurance policies were promulgated on “health insurance payment, off-site settlement, and financial compensation.” • All provinces provided support to Hubei Province. • Preschool preparation was improved. • Work resumed in phases and batches. • Health and welfare services were provided to returning workers in a targeted “one-stop” manner. • Normal social operations were restored in a stepwise fashion. • Knowledge about disease prevention was popularized to improve public health literacy and skills. • Comprehensive program of emergency scientific research is being carried out to develop diagnostics, therapeutics, and vaccines; delineate the spectrum of the disease; and identify the source of the virus.

^a^The information is provided from WHO report [6].

**Abbreviations:** COVID-19, coronavirus disease 19.

If these prevention and control measures were effective, the increasing trend of the daily number of new confirmed cases according to the onset date of illness (blue bars in Fig 2) should be halted and start to decline, forming a peak of this wave of the epidemic. However, this effect would not take place immediately and could only be expected after an incubation period (around 6 days). This is because those infected before January 20 could not be avoided and could continue to infect other people surrounding them in the following 6 days, pushing the peak to January 26. The real epidemic course responded exactly as predicted (blue bars in Fig 2), suggesting that the control efforts such as Wuhan’s lockdown were immediately effective. Since then, the daily number of local cases started to decline and continued until they totally disappeared recently.

### Intervention impact

How effective were the actions taken in China? A modeling study published in Lancet January 31, 2020 showed that if there were no reduction in transmissibility of the virus and the mobility of people and the previous momentum of the epidemic continued, the epidemic in Wuhan would peak around late April 2020 with some 30,000 new cases daily. The epidemic in Wuhan actually peaked February 4, 2020, with 1,967 new confirmed cases reported that day (yellow bars in Fig 2). This suggests that the control efforts in China since January 20 may have suppressed the peak number of confirmed cases by over 93.4% and also ended the entire epidemic ahead of the predicted time by approximately 2 months [38]. An article in Science drew a similar conclusion that travel restriction in Wuhan might have reduced 80% of cases outside of China before March 2020 [39]. A more recent Susceptible, Exposed, Infectious, Removed (SEIR) modeling study from Wuhan showed interventions had prevented 94.5% of infections [40].

On March 18, there were 34 cases reported, as compared with 3,893 on the peak day on February 4, representing a 99.1% reduction. All the 34 cases were in fact imported. Since February 9, China has been gradually resuming normal living and working order. The steadily declining trend since then suggests a rebound is unlikely to occur (yellow bars in Fig 2), although China now faces an increasing risk of imported epidemic (Fig 2).

While the epidemic is abating in China, it has taken off outside the country (Fig 2). From February 18 to March 18, the total number of cases in developed countries has jumped from 256 to 86,894, a 339-fold increase or a daily growth rate of 21.4%, very similar to the situation before 20 January 2020 in China. However, a question is raised as to why the epidemic in less developed countries (excluding China) was increasing at a rate only 26.0% that in developed countries during the same period (Fig 2).

## Transmission

### Routes of transmission

Transmission now is entirely from human to human, but the mechanisms of transmission are less conclusive. Close contact via droplets and fomites are common transmission routes.

However, the virus’ RNA was commonly detected in bronchoalveolar lavage fluids [2,41–43], pharyngeal swabs [14,22,24,31,44–47], nasal swabs [41,42,45,46], sputum [22,41,42], saliva [41,48], lung tissue [41], and rectal swabs [14,41,42,46,47,49,50] and sometimes also in conjunctival swabs [51], blood [14,41,42,46,52], and urine [41,42,46]. The virus was also successfully isolated through culture from bronchoalveolar lavage fluids [3], saliva [48], urine [53], and rectal specimens [53]. Furthermore, the RNA could persist for as long as 37 days in adults and 51 days in children [32,47]. These findings suggest that the virus can be shed through multiple outlets and other routes of transmission are also possible.

Furthermore, a study in Singapore showed that the virus’ RNA was detectable on various surfaces of rooms where infected patients were quarantined [54]. An American study showed that the virus can survive for 3.0 hours in aerosols and 2–3 days on plastics and metals [55]. These studies raised the possibility of airborne and fecal–mouth transmission. However, such routes of transmission are unlikely common in general public places because thousands of samples from a large range of public environments in Guangzhou failed to show any evidence of the virus [56].

### Clustering outbreaks

A prominent feature of COVID-19 is clustering, in particular outbreaks in families, making close contact the most common mechanism of transmission [6,34,57,58]. It was reported that familial clusters contributed up to 78%–85% of confirmed cases in China [6]. An analysis of 377 clusters involving 1,719 cases showed that familial outbreaks accounted for 79% of all clusters, with each causing a median number of 4 cases. Other types of clusters included dining gatherings (10%, each with a median of 5 cases), shopping malls and supermarkets (6%, 13), workplace gatherings (3%, 6), and public transport vehicles (2%, 6) [34]. Clusters in prisons were also reported, with 34–230 cases in an outbreak [59].

Familial contacts are also a high-risk exposure. In general, 1%–5% would be infected after close contact with patients or travel to Hubei epidemic areas [6]. A more recent follow-up study of 4,950 persons with close contact with patients showed the infection risk of high-frequent household contact was as high as 15.8%, as compared with only 1.0% in healthcare settings and 0.1% for patients taking contaminated public transports [60]. Household outbreaks accounted also for 83.7% of cases caused through all transmission forms.

However, the predominance of familial clustering by no means excludes the possibility of effective transmission in the absence of identifiable close contacts. For example, from January 14 to February 18, a series of 40 cases were found associated with a shopping mall in Baodi District of Tianjin, China, which were 31.3% of all the cases in the city [30]. Of the 40 cases, 6 (15.0%) were shop employees and 19 (47.5%) customers, who further caused 15 (37.5%) secondary cases outside the shopping mall. The first case was a shop employee who infected another 5 employees working in adjacent areas mostly with no close-contact opportunities. It is thus postulated that 5 infected employees and 19 infected customers were possibly infected through occasional contacts or even aerosols.

This was further evidenced in the outbreak in the 3,711 passengers on the Diamond Princess cruise ship. The first patient left the ship on January 25 and was diagnosed February 1. Then, the entire ship was put under quarantine on the sea, but the number of new cases continued to rise. As of February 20, 634 cases had been diagnosed, including passengers and cruise staff [61]. Because quarantine was imposed and close contacts were limited, occasional contacts, aerosols, and even the central air conditioning with recirculating air were likely explanations of transmission for many cases.

### Transmission during incubation

Continuous nucleic-acid testing in those with close-contact history provided clear evidence that patients shed virus before symptoms appear in both adults and children, pointing to the possibility of transmission during incubation [47,61,62]. Studies also found the serial interval between the first and second generation of COVID-19 cases ranged between 4.0–4.6 days [34,63,64], which is shorter than the average incubation period of 6 days, providing further evidence for transmission during incubation [65].

Evidence is also available from observation of infections directly caused by patients in incubation, but cases were not common. For example, Lancet Infectious Disease reported a familial cluster of 11 cases caused by a case a week before symptoms appeared [66], in addition to other similar reports [67,68].

### Transmission from asymptomatic infection

Asymptomatic infection is a great concern for transmission because it is more difficult to discover and control. Asymptomatic infection is referred to infections with the virus that can be confirmed with nucleic-acid or serological testing and may cause radiological and laboratory changes but will never develop symptoms. Cases have been reported in both children and adults who were consecutively nucleic-acid positive but remained completely free of symptoms even 30 days after the last exposure [47,57,62], suggesting transmission from asymptomatic patients could be theoretically possible.

How often does asymptomatic infection exist in all the infected? A modeling study of the outbreak on the Diamond Princess cruise ship showed asymptomatic infection could, in theory, account for 17.9% of all infections [61]. Other modeling studies gave estimates as high as 30.8%–86.0% [40,62,69]. This is, however, in sharp contrast to the empirical observation that only 1.0% were diagnosed purely via viral nucleic-acid testing and completely asymptomatic [70]. Close follow-up of 4,950 close contacts showed 8 (6.2%) of the 129 cases were completely asymptomatic by the end of 14 days’ quarantine [60].

These large different estimates may partly be explained by different definitions for asymptomatic infection, rigorousness in assessing symptoms, and the populations studied. As of March 18, some 700,000 close contacts had been identified and clinically closely observed in China. If 5% of them eventually contracted infection, it was impossible that there were only less than 1,000 asymptomatic patients in the entire country.

Cases infected from asymptomatic infection have also been reported but not common. A follow-up study of 24 patients asymptomatic at diagnosis in Nanjing found 7 were completely asymptomatic throughout, with 1 having caused a familial outbreak [71]. Furthermore, a Journal of the American Medical Association (JAMA) article reported a patient caused a familial cluster of 5 cases but remained asymptomatic and free of any radiological and laboratory alterations for over 19 days when observation ended [72].

How often does asymptomatic infection exist in general populations? In Guangdong, it was found the RNA evidence of SARS-CoV-2 was positive in only 48 in 190,000 outpatients of fever clinics and 3 in 60,000 Hubei returnees [6, 60], suggesting asymptomatic infections be even rarer in general populations, although Hubei may be higher. Large serological studies have yet to be conducted.

In summary, infection via asymptomatic infection is possible but unlikely a common mechanism of transmission. Otherwise, many close contacts would be untraceable, and the epidemic could have not been effectively contained as it is in China and Korea largely by finding and quarantining close contacts through identified patients.

### Transmission during convalescence

There is some evidence for persistent positivity for the virus’ nucleic acid in throat swabs and in particular rectal swabs after recovery, which could last as long as 37 to 51 days in adults [32,47]. Interesting cases were reported in a JAMA article that 4-time consecutive-test–negative convalescent patients became positive again 5–13 days later [64]. These findings suggest patients could potentially shed the virus during convalescence but should not be considered direct evidence of replication-competent viruses that can cause further infections. We have not seen reports of people infected by patients in the convalescent period.

## Diagnosis

Finding patients early is crucial for control of the epidemic. Because the clinical manifestations of COVID-19 are nonspecific, a definitive diagnosis largely relies on nucleic-acid testing [73,74]. Nucleic-acid testing should be performed on throat swabs and/or rectal swabs in particular during convalescence. Two consecutive samples 48 hours apart can considerably improve sensitivity. Although the nucleic-acid testing provided reliable evidence of infection, evidence of nucleic acid does not necessarily indicate the presence of infectious virus, and a negative result does not necessarily rule out the diagnosis. For highly suspected patients, chest computerized tomography (CT) should be used to supplement nucleic-acid testing. Serum immunoglobulin M (IgM) and IgG tests are now available and can help boost up the detection rate [75] but will be less useful for initiation of treatment and quarantine.

During an epidemic of a highly infectious disease, diagnostic methods with a high sensitivity are preferable, or we may delay both treatment and isolation and cause undesirable consequences. Until February 12, the diagnostic criteria China used required throat swab test be positive by a real-time reverse transcription polymerase chain reaction (RT-PCR). First, this requirement delayed diagnosis of patients for at least 5 days at the early days of the epidemic because of an insufficient supply of testing kits. Second, it had missed many true patients because of the low sensitivity (about 30%–50%) and caused delays in treatment and quarantine [76]. The low sensitivity may have resulted from patients who are not shedding virus or are shedding virus at an undetectable amount in early infection, convalescence, or a silent intermittent-shedding period. The low sensitivity can also be caused by inappropriate methods of collecting samples, sampling sites such as throat swabs instead of bronchoalveolar lavage fluid, and storage and transport of specimens.

The low sensitivity of nucleic-acid testing later turned out to be a serious problem in the epicenter Hubei Province, in particular in Wuhan. Studies found that in people highly suspected to have COVID-2019, chest CT evidence of viral pneumonia often preceded PCR evidence and appeared in 97% of early infections [77.^78^]. Consequently, China had to revise the diagnostic criteria so as to include all clinically suspected patients [74]. The new criteria were used only for 4 days between February 12–15. As a result, a total of 18,431 patients (excluded in Fig 2) were clinically diagnosed, hospitalized, and treated free of cost and put under quarantine. This helped greatly in clearing up the potential sources of infection in the population. A lesson learned is that heavy reliance on viral evidence for diagnosis gained in accuracy but caused delays in quarantine and treatment.

## Clinical features

COVID-19 is a new infectious disease, and all are susceptible. Of the 44,672 confirmed cases before February 11, 2020, patients were between the age of 30–79 years [70]. The majority were diagnosed in Hubei Province (75%), and most early cases reported Wuhan-related exposures (86%). Eighty-one percent were mild or moderate cases (for having mild pneumonia or lack of pneumonia). However, 14% were severe (for having dyspnea, respiratory frequency 30/min, blood oxygen saturation 93%, partial pressure of arterial oxygen to fraction of inspired oxygen ratio <300, and/or lung infiltrates >50% within 24 to 48 hours), and 5% were critical (for having respiratory failure, septic shock, and/or multiple organ dysfunction or failure).

The overall case fatality was 2.3%. No deaths occurred in the group aged 9 years and younger, but the fatality was 8.0% in cases aged 70–79 years and 14.8% in those aged 80 years and older. No deaths were reported among mild, moderate, or severe cases; all deaths occurred in critical cases, showing a fatality of 49.0%. The fatality was elevated among those with preexisting comorbidities: 10.5% for cardiovascular disease, 7.3% for diabetes, 6.3% for chronic respiratory disease, 6.0% for hypertension, and 5.6% for cancer. Fatality also differed considerably by area [70]. As of March 19, fatality was 5.0% (2,498/50,005) in Wuhan, 3.6% (634/17,759) in Hubei excluding Wuhan, and 0.88% (116/13,167) in China excluding Hubei [5]. In contrast, in Italy, 12% of all detected COVID-19 cases and 16% of all hospitalized patients were admitted to the intensive care unit, and the case fatality was 5.8%, double that in China [79]. In addition, high Sequential Organ Failure Assessment (SOFA) score and d-dimer were also found related to poor prognoses [32].

Table 3 summarizes clinical, radiological, and laboratory features of 2019-nCoV patients at admission reported in 5 major relevant studies. A large number of patients also had common morbidities like cardiovascular diseases, hypertension, and diabetes. Common symptoms included fever, cough, dyspnea, sputum, myalgia, fatigue, and anorexia. Most patients showed radiological abnormalities, but only 20% demonstrated ground-glass opacities. Some patients showed alternations in lymphocyte counts, procalcitonin, aspartate aminotransferase (AST), and C-reactive protein. Acute respiratory distress syndrome (ARDS), acute kidney injury, acute cardiac injury, coinfections, and shock also existed but were not common.

**Table 3:** Demographics underlying medical conditions, symptoms and signs, laboratory findings, radiological alterations, treatments, complications, and prognoses with COVID-19 from 5 studies of 1,439 patients in China^a^.

Variables	Studies				
Authors	Huang and colleagues [14]	Chen and colleagues [44]	Wang and colleagues [31]	Guan and colleagues [24]	Xu and colleagues [22]
**Study period**	Dec 16, 2019 to Jan 2, 2020	Jan 1, 2020 to Jan 20, 2020	Jan 1, 2020 to Jan 28, 2020	Dec 11, 2019 to Jan 29, 2020	Jan 10, 2020 to Jan 26, 2020
**Study area**	Wuhan	Wuhan	Wuhan	China	Zhejiang
**Hospitals involved**	1 hospital	1 hospital	1 hospital	552 hospitals	7 hospitals
**Number of patients**	41	99	138	1,099	62
**Characteristics (n [%], n/N [%], mean [SD or median [IQR])**					
Age (years)	49.0 (41.0–58.0)	55.5 (13.1)	56.0 (42.0–68.0)	47.0 (35.0–58.0)	41 (32–52)
≥65 years	6 (14.6%)	NA	NA	153/1,011 (15.1%)	NA
Males	30 (73.2%)	67 (67.7%)	75 (54.3%)	637/1,096 (58.1%)	35 (56.5%)
Nosocomial infection	NA	NA	57 (41.3%)	NA	NA
Healthcare worker	NA	NA	40 (29.0%)	NA	NA
Current smoking	3 (7.3%)	NA	NA	137/1,085 (12.6%)	NA
**Symptoms and signs (n [%] or n/N [%])**					
Fever	40 (97.6%)	82 (82.8%)	136 (98.6%)	473/1,081 (43.8%)	48 (77.4%)
Cough	31 (75.6%)	81 (81.8%)	82 (59.4%)	745 (67.8%)	50 (80.6%)
Dyspnea	22/40 (55.0%)	31 (31.3%)	43 (31.2%)	205 (18.7%)	NA
Sputum production	11/39 (28.2%)	NA	37 (26.8%)	370 (33.7%)	35 (56.5%)
Myalgia	18^f^	11 (11.1%)	48 (34.8%)	164 (14.9%)	NA
Fatigue	NA	NA	96 (69.6%)	419 (38.1%)	NA
Headache	3/38 (7.9%)	8 (8.1%)	9 (6.5%)	150 (13.6%)	21 (33.9%)
Diarrhea	1/38 (2.6%)	2 (2.0%)	14 (10.1%)	42 (3/8%)	3 (4.8%)
Sore throat or pharyngalgia	NA	5 (5.1%)	24 (17.4%)	153 (13.9%)	NA
Nausea or vomiting	NA	1 (1.0%)	NA	55 (5.0%)	NA
Respiratory rate >24 breaths per minute	12 (29.3%)	NA	NA	NA	2 (3.2%)
Anorexia	NA	NA	55 (39.9%)	NA	NA
Chills	NA	NA	NA	126 (11.5%)	NA
**Comorbidities (n [%])**					
Any comorbidity	13 (31.7%)	50 (51.5%)	64 (46.4%)	261 (23.7%)	20 (32.3%)
Cardiovascular disease	6 (14.6%)	40 (40.4%)	20 (14.5%)	27 (2.5%)	NA
Hypertension	6 (14.6%)	NA	43 (31.2%)	165 (15.0%)	5 (8%)
Diabetes	8 (19.5%)	NA	14 (10.1%)	81 (7.4%)	1 (1.6%)
Respiratory disease	1 (2.4%)	1 (1.0%)	4 (2.9%)	12 (1.1%)	1 (1.6%)
Malignancy	1 (2.4%)	1 (1.0%)	10 (7.2%)	10 (0.9%)	NA
Chronic kidney disease	NA	NA	4 (2.9%)	8 (0.7%)	1 (1.6%)
Chronic liver disease	1 (2.4%)	NA	4 (2.9%)	23 (2.1%)	7 (11.3%)
**Laboratory findings (normal range; n [%], n/N [%], mean [SD] or median [IQR])**					
White blood cell count (4.0–10.0 × 10^9^/L)	6.2 (4.1–10.5)	7.5 (3.6)	4.5 (3.3–6.2)	4.7 (3.5–6.0)	4.7 (3.5–5.8)
White blood cell count < 4.0 × 10^9^/L	10/40 (25.0%)	<3.5:9 (9.1%)	NA	330/978 (33.7%)	19 (30.6%)
Neutrophil count (1.8–6.3 × 10^9^/L)	5.0 (3.3–8.9)	5.0 (3.3–8.1)	3.0 (2.0–4.9)	NA	2.9 (2.0–3.7)
Lymphocyte count (1.1–3.2 × 10^9^/L)	0.8 (0.6–1.1)	0.9 (0.5)	0.8 (0.6–1.1)	1.0 (0.7–1.3)	1.0 (0.8–1.5)
Lymphocyte count < 1.0 × 10^9^/L	26 (63.4%)	<1.1:35 (35.4%)	NA	<1.5:731/879 (83.2%)	26 (41.9%)
Platelet count (125.0–350.0 × 10^9^/L)	164.5 (131.5–263.0)	213.5 (79.1)	163.0 (123.0–191.0)	168.0 (132.0–207.0)	176.0 (135.8–215.5)
Platelet count < 100.0 × 10^9^/L	2/40 (5.0%)	<125.0:12 (12.1%)	NA	<150.0:315/869 (36.2%)	3 (4.8%)
APTT (21.0–37.0 s)	27.0 (24.2–34.1)	27.3 (10.2)	31.4 (29.4–33.5)	NA	NA
PT (10.5–13.5 s)	11.1 (10.1–12.4)	11.3 (1.9)	13.0 (12.3–13.7)	NA	NA
D-dimer (0.0–0.5 mg/L)	0.5 (0.3–1.3)	0.9 (0.5–2.8)	0.2 (0.1–0.4)	≥0.5:260/560 (46.4%)	0.2 (0.2–0.5)
Creatine kinase (50.0–310.0 U/L)	132.5 (62.0–219.0)	85.0 (51.0–184.0)	92.0 (56.0–130.0)	NA	69.0 (40.5–101.0)
Creatine kinase > 185.0 U/L	13/40 (32.5%)	>310.0:13 (13.1%)	NA	≥200:90/657 (13.7%)	5 (8.1%)
Albumin (40.0–55.0 g/L)	31.4 (28.9–36.0)	31.6 (4.0)	NA	NA	NA
ALT (9.0–50.0 U/L)	32.0 (21.0–50.0)	39.0 (22.0–53.0)	24.0 (16.0–40.0)	>40:158/741 (21.3%)	22 (14–34)
AST (15.0–40.0 U/L)	34.0 (26.0–48.0)	34.0 (26.0–48.0)	31.0 (24.0–51.0)	NA	26 (20–32)
AST ≥ 40.0 U/L	15 (36.6%)	35 (35.4%)	NA	168/757 (22.2%)	10 (16.1%)
Total bilirubin (5.0–21.0 μmol/L)	11.7 (9.5–13.9)	15.1 (7.3)	9.8 (8.4–14.1)	>17.1:76/722 (10.5%)	NA
Lactate dehydrogenase (120.0–250.0 U/L)	286.0 (242.0–408.0)	336.0 (260.0–447.0)	261.0 (182.0–403.0)	NA	205.0 (184.0–260.5)
Lactate dehydrogenase > 245.0 U/L	29/40 (72.5%)	75 (75.8%)	NA	≥250.0:277/675 (41.0%)	17 (27.4%)
Hemoglobin (113.0–151.0 g/L)	126.0 (118.0–140.0)	129.8 (14.8)	NA	134.0 (119.0–148.0)^c^	137.0 (128.8–152.3)
Potassium (3.5–5.3 mmol/L)	4.2 (3.8–4.8)	NA	NA	3.8 (3.5–4.2)^d^	3.7 (3.5–3.9)
Sodium (137.0–147.0 mmol/L)	139.0 (137.0–140.0)	NA	NA	138.2 (136.1–140.3)^e^	139 (127–141)
Creatinine (57.0–111.0 μmol/L)	74.2 (57.5–85.7)	75.6 (25.0)	72 (60–87)	NA	72.0 (61.0–84.0)
Creatinine > 133.0 μmol/L	4 (9.8%)	>111.0:3 (3.0%)	NA	12/752 (1.6%)	3 (4.8%)
Blood urea nitrogen (3.6–9.5 mmol/L)	NA	5.9 (2.6)	NA	NA	NA
Hypersensitive troponin (0.0–26.2 pg/mL)	3.4 (1.1–9.1)	NA	6.4 (2.8–18.5)	NA	NA
Myoglobin (0.0–146.9 ng/mL)	NA	49.5 (32.2–99.8)	NA	NA	NA
Glucose (3.9–6.1 mmol/L)	NA	7.4 (3.4)	NA	NA	NA
Interleukin-6 (0.0–7.0 pg/mL)	NA	7.9 (6.1–10.6)	NA	NA	NA
Erythrocyte sedimentation rate (0.0–15.0 mm/h)	NA	49.9 (23.4)	NA	NA	NA
Serum ferritin (21.0–274.7 ng/mL)	NA	808.7 (490.7)	NA	NA	NA
Procalcitonin (0.0–0.05 ng/mL)	0.1 (0.1–0.1)	0.5 (1.1)	NA	NA	0.04 (0.03–0.06)
Procalcitonin ≥ 0.1 ng/mL	12/39 (30.8%)	>5.0: 6 (6.1%)	≥0.05:49 (35.5%)	≥0.5:35/633 (5.5%)	7 (11.3%)
C-reactive protein (0.0–5.0 mg/L)	NA	51.4 (41.8)^b^	NA	NA	NA
C-reactive protein > 5.0 mg/L	NA	63/73 (86.3%)	NA	≥10:481/793 (60.7%)	NA
Systolic pressure (mmHg)	125.0 (119.0–135.0)	NA	NA	NA	97 (87–106)
**Radiologic findings (n [%] or n/N [%])**					
Chest X-ray or CT					
Unilateral pneumonia	NA	25 (25.3%)	NA	NA	NA
Bilateral pneumonia	40 (97.6%)	74 (74.7%)	138 (100.0%)	NA	52 (83.9%)
Multiple mottling and ground-glass opacity	NA	14 (14.1%)	NA	NA	NA
Chest X-ray	NA	NA	NA	162/274 (59.1%)	NA
Ground-glass opacity	NA	NA	NA	55/274 (20.1%)	NA
Local patchy shadowing	NA	NA	NA	77/274 (28.1%)	NA
Bilateral patchy shadowing	NA	NA	NA	100/274 (36.5%)	NA
Interstitial abnormalities	NA	NA	NA	12/274 (4.4%)	NA
Chest CT	NA	NA	NA	840/975 (86.2%)	NA
Ground-glass opacity	NA	NA	NA	550/975 (56.4%)	NA
Local patchy shadowing	NA	NA	NA	409/975 (41.9%)	NA
Bilateral patchy shadowing	NA	NA	NA	505/975 (51.8%)	NA
Interstitial abnormalities	NA	NA	NA	143/975 (14.7%)	NA
**Treatments (n (%))**					
Antiviral treatment	38 (92.7%)	75 (75.8%)	124 (89.9%)	393 (35.8%)	55 (88.7%)
Antibiotic treatment	41 (100.0%)	70 (70.7%)	138 (100.0%)	637 (58.0%)	28 (45.2%)
Antifungal treatment	NA	15 (15.2%)	NA	31 (2.8%)	NA
Corticosteroid treatment	9 (22.0%)	19 (19.2%)	62 (44.9%)	204 (18.6%)	16 (25.8%)
CRRT	3 (7.3%)	9 (9.1%)	2 (1.4%)	9 (0.8%)	NA
IVIg therapy	NA	27 (27.3%)	NA	144 (13.1%)	NA
Noninvasive mechanical ventilation	10 (24%)	13 (13.1%)	15 (10.9%)	56 (5.1%)	NA
Invasive mechanical ventilation	2 (4.9%)	4 (4.0%)	17 (12.3%)	25 (2.3%)	NA
ECMO	2 (4.9%)	3 (3.0%)	4 (2.9%)	5 (0.5%)	NA
Oxygen therapy	41 (100.0%)	75 (75.8%)	106 (76.8%)	454 (41.3%)	NA
**Complications (n (%))**					
ARDS	12 (29.3%)	17 (17.2%)	27 (19.6%)	37 (3.4%)	1 (1.6%)
Acute kidney injury	3 (7.3%)	3 (3.0%)	5 (3.6%)	6 (0.5%)	NA
Acute cardiac injury	5 (12.2%)	NA	10 (7.2%)	NA	NA
Co- or secondary infection	4 (9.8%)	5 (5.1%)	NA	NA	NA
Shock	3 (7.3%)	4 (4.0%)	12 (8.7%)	12 (1.1%)	NA
ICU unit admission	13 (31.7%)	23 (23.2%)	36 (26.1%)	55 (5.0%)	1 (1.6%)
**Prognoses (n (%))**					
Hospitalization	7 (17.1%)	57 (57.6%)	85 (61.6%)	1,029 (93.6%)	61 (98.4%)
Discharge	28 (68.3%)	31 (31.3%)	47 (34.1%)	55 (5.0%)	1 (1.6%)
Death	6 (14.6%)	11 (11.1%)	6 (4.3%)	15 (1.4%)	0 (0.0%)

**Abbreviations:** ALT, alanine aminotransferase; APTT, activated partial thromboplastin time; ARDS, acute respiratory distress syndrome; AST, aspartate aminotransferase; COVID-19, coronavirus disease 19; CRRT, continuous renal replacement therapy; CT, computerized tomography; ECMO, extracorporeal membrane oxygenation; ICU, intensive care unit; IVIg, intravenous immunoglobulin; NA, not available; PT, prothrombin time.

^a^The information provided in the table is from Hang and colleagues [14], Chen and colleagues [44], Wang and colleagues [31], Guan and colleagues [24], and Xu and colleagues [22].

^b^Data on C-reactive protein were missing for 26 patients (26.3%).

^c^Data on hemoglobin were missing for 226 patients (20.6%).

^d^Data on potassium were missing for 349 patients (31.8%).

^e^Data on sodium were missing for 363 patients (33.0%).

^f^Data on myalgia or fatigue were missing for 18 patients in total.

No antiviral agents have been approved by regulatory agencies for the treatment of COVID-19, and the clinical management of the infection was supportive care. Antiviral drugs, antibiotic treatments, Chinese herbal medicine, and corticosteroids were commonly used, and extracorporeal membrane oxygenation (ECMO) was applied to less than 5% (Table 3), but their effectiveness is largely unknown. Antimicrobial drugs were recommended based on their effectiveness in preventing infections before exposure or after exposure to microbial pathogens and in reducing the risk of secondary spread of infection [80]. A recent clinical trial showed that in hospitalized adult patients, no additional benefit on major outcomes was observed from lopinavir–ritonavir on top of standard care [81]. Against general evidence of no effect in treating respiratory infection due to respiratory syncytial virus (RSV), influenza, SARS-CoV, or MERS-CoV [82], corticosteroids were commonly used in treating COVID-19.

Overall, considering the clinical features of the disease, it is advisable that mild or moderate patients be cared for and quarantined at home when hospital resources are tight.

## Miscellaneous and lessons learned

### Infections in children

Children are believed to be less likely to be infected. A report of the earliest 425 cases found no children under the age of 15 years were infected [21]. This is sharply disproportionate to the percentage of children of the same age band (17.8%) in the Chinese general population [83]. A more recent report showed that 2.2% of the confirmed cases (965/44,672) were under the age of 20 years as compared to 24.1% of children of this age band in the general population [70]. Furthermore, a comparison study showed that the infection rate was 3.5% (111/3,174) in exposed adults and 1.3% (10/745) in exposed children, suggesting if exposed, adults are 2.7 more likely to be infected than children (*P* = 0.002) [47]. If infected, compared with adult patients, pediatric cases in general are clinically mild and less likely to show typical radiological alterations in the lungs [47,84–86].

Sparse data are available on intrauterine or perinatal transmission. In 2 reports including a total of 18 pregnant women with suspected or confirmed COVID-19 pneumonia, there was no molecular evidence of transmission from mother to neonate. In 2 infected neonatal cases documented so far [87], one was diagnosed on the seventeenth day of birth and was likely infected because of close contact with the mother and maternity matron, both of whom were infected before the child was born [88]. The other case was diagnosed 36 hours after birth; the source and time of transmission were unclear.

### Infections in health workers

Among the first 44,672 cases, a total of 1,716 were health workers (3.8%), 1,080 of whom were in Wuhan (63%), of which 40% of the infections occurred in hospitals and the rest in general communities [6,70]. Interestingly, none of the infections occurred in the 42,000 physicians who were sent out to support Hubei. Overall, 14.8% of confirmed cases among health workers were classified as severe or critical, and 5 died [70].

The outbreaks in health workers at the early stage of SARS and COVID-19 epidemics are a feature in the 2 epidemics, suggesting that alertness to infectious diseases and protection is not sufficiently high among clinicians at the early stage of the epidemic. They are also partly because hospitals were in general unprepared for highly contagious infectious diseases and taking care of a large increase in the number of patients. For example, as the pandemic accelerated, access to personal protective equipment for health workers was a problem. Some medical staff were waiting for equipment while already seeing patients who might be infected or were supplied with equipment that might not meet requirements [89]. Furthermore, during the peak period, a huge number of fever patients poured into outpatient departments of hospitals and made them a major place for spreading the virus. On the other hand, hospitals incapable of quickly admitting all the patients left thousands of them in the communities and increased the risk of transmission. The situation was alleviated after some 60,000 hospital beds were urgently created in Wuhan and 42,000 doctors from other provinces were sent out to Hubei.

### Vaccine or quarantine?

What we believe largely determines how we act. The remarks of Dr. Bruce Aylward, WHO’s Secretary General, on February 24 [90] may reflect what we believe today in confronting this new deadly disease: “In the world of preparedness and planning, I suffer the same biases as or maybe error of thinking as many people… we don’t have a vaccine, we don’t have a therapeutic.” How can we defeat this epidemic?

Indeed, it was believed that actions were not sufficiently fast at the early stage of the epidemic in China, which may partly be a result of our societal belief in new scientific methods such as confirming the pathogen via virologic diagnostics, antiviral drugs, and vaccines. However, the success of these research efforts is not immediately foreseeable but will compete for the public’s attention and resources with the epidemiological and clinical investigations that are immediately important for formulating preventive and treatment policies. For example, the specific viral diagnostic testing kits for 2019-nCoV were already available January 12, but whether or not the virus could transmit among humans was still debated until February 20. Later, the PCR method tended to miss diagnoses and, in a sense, facilitated the spread of the disease.

## Conclusions

COVID-19 is highly contagious but can be effectively contained with quick and determined traditional nonpharmacological measures such as isolation and social distancing, as is demonstrated in China and Korea. The time window for critical early action may be missed if we hold that a large number of asymptomatic or undocumented infections exist to facilitate the rapid dissemination of infection so that the epidemic is beyond control. Given the increasing momentum of the pandemic, millions of infections and many deaths could soon occur if control measures were not put in order in time and with resolve.
