# Correction: SARS-CoV-2 infection in China—Before the pandemic

**DOI:** 10.1371/journal.pntd.0010520

**Published:** 2022-06-06

**Authors:** Huiying Liang, Lingling Zheng, Huimin Xia, Jinling Tang

The legends of Fig 1 and Fig 2 are in the correct location, but the images are reversed in order. Please see the correctly ordered Fig 1 and Fig 2 below.

**Fig 1:**
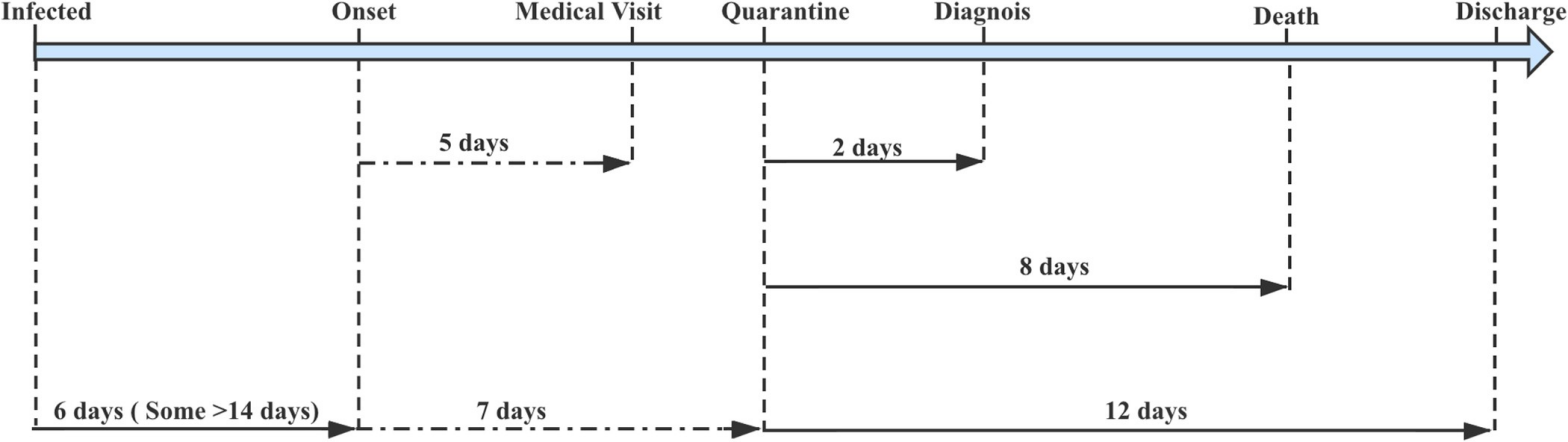
Approximate number of days from infection to onset, to first medical visit, to hospitalization, to laboratory diagnosis, and to discharge with COVID-19 patients.

**Fig 2:**
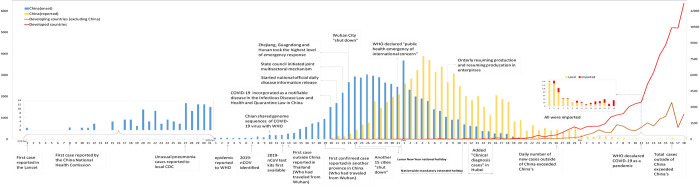
Epidemic curve of the confirmed cases of COVID-19 and time points of major related events.

(Table 1 is referred for more details.) CDC, Centers for Disease Control and Prevention; COVID-19, coronavirus disease 19; 2019s-nCoV, 2019 novel coronavirus.

Daily numbers of confirmed cases are plotted in bars by the date of onset (blue) and by that of diagnosis/reporting (yellow). In the inset are cases in December 2019 (left) and after March 5, 2020 (right). Cases reported outside of China were separately plotted between western countries (red lines) and other countries (brown lines). The daily number of cases by the date of onset were adapted from the data in Fig 3 in the Report of the WHO–China Joint Mission on COVID-19 [6], the rest of the data are from China CDC [5], and the events are from various publications included in this paper. CDC, Centers for Disease Control and Prevention; COVID-19, coronavirus disease 19.
